# Alzheimer’s Disease Among American Minority Populations: An Ecological Exploratory Study

**DOI:** 10.1093/geroni/igab046.1350

**Published:** 2021-12-17

**Authors:** Maria LaQuaglia, Marina Celly Martins Ribeiro de Souza, Carolina Marques Borges

**Affiliations:** 1 The College of New Jersey, Ewing , New Jersey, United States; 2 The College of New Jersey, The College of New Jersey, New Jersey, United States

## Abstract

A significant public health concern with regards to increasing rates of Alzheimer’s is that it disproportionately affects minority groups in the United States. The present ecological exploratory study uses secondary aggregate data from the fifty United States in the year of 2019. The purpose of this study was to address the disparities in Alzheimer’s in minority populations in the US and explore associated factors. The “minority” populations considered were African American and Latino populations, and the “majority” population was referred to as “white”. The data was extracted from the United States Census Bureau, the CDC National Center for Health Statistics, and the Behavioral Risk Factor Surveillance System (BRFSS) Dataset. The prevalence rates of Alzheimer’s disease are greatest in both older Latinos (12.2%) and African Americans (13.8%), compared to older whites (10.3%) in the investigated time period. Our results showed that being over 65 years old (p=.009), with a below-average ($62,843) median household income (p=.024), history of stroke (p=.029), and being a part of the Latino population (p=.036), were significantly associated with Alzheimer’s mortality rates in the United States. By identifying disparities in access to Alzheimer’s healthcare and at-risk communities, more comprehensive intervention strategies can be developed to promote change and advocate for more Alzheimer’s education and resource allocation for minority populations.

